# The role of vitamin D in outcomes of critical care in COVID-19 patients: evidence from an umbrella meta-analysis of interventional and observational studies

**DOI:** 10.1017/S1368980024000934

**Published:** 2024-04-24

**Authors:** Abdolreza Jamilian, Faezeh Ghalichi, Fatemeh Hamedi Kalajahi, Nima Radkhah, Neda Jourabchi, Vali Musazadeh, Ehsan Amini-Salehi, Meysam Zarezadeh, Alireza Ostadrahimi

**Affiliations:** 1 City of London Dental School, University of Bolton, London, UK; 2 Orthodontic Department, Dental School, Tehran Medical Sciences, Islamic Azad University, Tehran, Iran; 3 Student Research Committee, Tabriz University of Medical Sciences, Tabriz, Iran; 4 Department of Clinical Nutrition, School of Nutrition and Food Sciences, Tabriz University of Medical Sciences, Tabriz, Iran; 5 School of Medicine, Guilan University of Medical Sciences, Rasht, Iran; 6 Nutrition Research Center, School of Nutrition and Food Sciences, Tabriz University of Medical Sciences, Attar-Neishaburi St., Golgasht Alley, Azadi Blvd., Tabriz, Iran

**Keywords:** Vitamin D, Mortality, Intensive care unit, Critical illness, COVID-19, Umbrella meta-analysis

## Abstract

**Objectives:**

Several meta-analyses have suggested the beneficial effect of vitamin D on patients infected with severe acute respiratory syndrome coronavirus-2. This umbrella meta-analysis aims to evaluate influence of vitamin D supplementation on clinical outcomes and the mortality rate of COVID-19 patients.

**Design:**

Present study was designed as an umbrella meta-analysis. The following international databases were systematically searched till March 2023: Web of Science, PubMed, Scopus, and Embase.

**Settings:**

Random-effects model was employed to perform meta-analysis. Using AMSTAR critical evaluation tools, the methodological quality of the included meta-analyses was evaluated.

**Participants:**

Adult patients suffering from COVID-19 were studied.

**Results:**

Overall, 13 meta-analyses summarising data from 4 randomised controlled trial and 9 observational studies were identified in this umbrella review. Our findings revealed that vitamin D supplementation and status significantly reduced mortality of COVID-19 [Interventional studies: (ES = 0·42; 95 % CI: 0·10, 0·75, *P* < 0·001; *I* ^2^ = 20·4 %, *P* = 0·285) and observational studies (ES = 1·99; 95 % CI: 1·37, 2·62, *P* < 0·001; *I* ^2^ = 00·0 %, *P* = 0·944). Also, vitamin D deficiency increased the risk of infection and disease severity among patients.

**Conclusion:**

Overall, vitamin D status is a critical factor influencing the mortality rate, disease severity, admission to intensive care unit and being detached from mechanical ventilation. It is vital to monitor the vitamin D status in all patients with critical conditions including COVID patients.

The severe acute respiratory syndrome coronavirus-2 caused a novel pandemic named coronavirus disease 19 (COVID-19). Severe acute respiratory syndrome coronavirus-2 generates an inflammatory status and induces the production of C-reactive protein, d-dimer, IL-6, etc. which could lead to acute distress syndrome especially in the second week due to cytokine storm^(1)^. Besides auxiliary drugs to treat and reduce the complications of COVID-19 such as corticosteroids, no proven drugs have been generated yet and the search for current available medications has been prioritised.

Vitamin D is a vital component in modulation of the immunological response in both infectious and autoimmune diseases in different ways^(2)^. A substantial body of evidence indicates that active form of vitamin D (1,25 dihydroxy vitamin D) is essential for the modulation of innate and adaptive immunity (T-lymphocyte activation and B lymphocyte proliferation)^(3)^ and reduces the risk of cytokine storm and pro-inflammatory markers^(4)^ and maintenance of pulmonary barrier integrity^(5)^. In case of vitamin D deficiency, these mechanisms will fail and make host vulnerable to different types of infections such as respiratory diseases. Several studies now support that vitamin D sufficiency has a beneficial effect on acute respiratory tract infections^(6–8)^ and attenuates the risk of respiratory tract infections. Initially, it was indicated that vitamin D deficiency could lead to higher mortality rates, longer stay in intensive care unit (ICU), higher mechanical ventilation rate and its severity. Hence, during the pandemic, vitamin D attracts an attention on COVID-19 treatment and its complications.

Relationship between vitamin D in COVID-19 outcomes is not based on solid evidence. High heterogeneity among the meta-analysis studies leads to dubious results on the effects of vitamin D on COVID-19 severity, and its complications and majority of the reviews remained inconclusive. Several meta-analyses have shown that vitamin D sufficiency and supplementation have a positive impact on COVID-19 outcomes^(9–12)^, while others did not support these results^(13–15)^. Therefore, present umbrella meta-analysis aimed to assess the role of vitamin D on clinical outcomes such as ICU admission, mechanical ventilation rate, severity and mortality in COVID-19-positive patients to provide valid and authentic evidence.

## Method and materials

Present umbrella meta-analysis has been developed according to the Preferred Reporting Items for Systematic Reviews and Meta-Analyses statement guidelines. The question of this study was based on PICO criteria: Participants (patients suffering from COVID-19), Intervention (vitamin D supplementation or status), Comparison (Control), Outcome (risk of infection, ICU admission, mechanical ventilation rate, severity and mortality).

### Search strategy and study selection

The scientific international databases including Web of Science, PubMed, Scopus and EMBASE were searched up to March 2023 to identify relevant studies. The search strategy was developed using the following MeSH and title/abstract keywords. The full search strategy for all databases is presented in see online supplementary material, Supplementary Table 1. The wild-card term ‘*’ was utilised to enhance the sensitivity of the search method. Also, the articles were confined to English language.

### Inclusion and exclusion criteria

Systematic reviews and meta-analysis studies investigating the effects of vitamin D were included in the current umbrella meta-analysis if they reported the effect of vitamin D on COVID-19 positivity status, severity, infection status, ICU admission, mechanical ventilation, prognosis including effect sizes (ES) and corresponding CI. In vitro, in vivo and ex vivo studies were excluded from this meta-analysis of meta-analyses.

### Quality assessment

The quality evaluation of the methodology of the included studies was examined by two reviewers (VM and FHK), using the AMSTAR^(16)^ independently. The AMSTAR questionnaire consists of 11 questions in which reviewers must respond with ‘yes’, ‘no’, ‘not applicable’ or ‘can’t answer’. Eleven is the highest score. Articles with a score of 7 or higher are regarded to be of good quality.

### Data extraction

Two independent reviewers (FHK and VM) screened the studies based on the eligibility criteria. In the first stage, the title and abstract were evaluated. Second, the full text of relevant papers was reviewed to determine whether the study could be included in the umbrella meta-analysis. All discrepancies were resolved by senior author’s decision (MZ).

Following data were ectracted: The year of publication, sample size, study location, study types, vitamin D deficiency definition, ES (weighted mean difference (WMD), standardised mean difference (SMD), odds ratio (OR), risk ratio (RR) and hazzard ratio (HR)) and CI for COVID-19 positivity status, severity, infection status, ICU admission and mortality, mechanical ventilation and mortality.

### Data synthesis and statistical analysis

The overall ES was calculated by combining the ES and CI for each included meta-analysis. A random-effects model was employed to perform the analysis. *I* ^2^ statistics and Cochrane’s *Q*-test were used to determine between-study heterogeneity; in the matter of *I* ^2^ value >50 % or *P* < 0·1 for the *Q*-test, it was regarded as significant heterogeneity. Sensitivity analysis was conducted to determine whether the overall ES was associated with the removal of one specific study from overall analysis. Begg’s test was used to assess publication bias. If the p-value for Begg’s test was <0·05, trim and fill analysis was carried out to adjust the publication bias. Stata software version 17·0 (Stata Corporation, College Station, TX, US) was used for all of the statistical analyses. *P* < 0·05 was considered as significant level.

## Results

### Systematic review

**Fig. 1:**
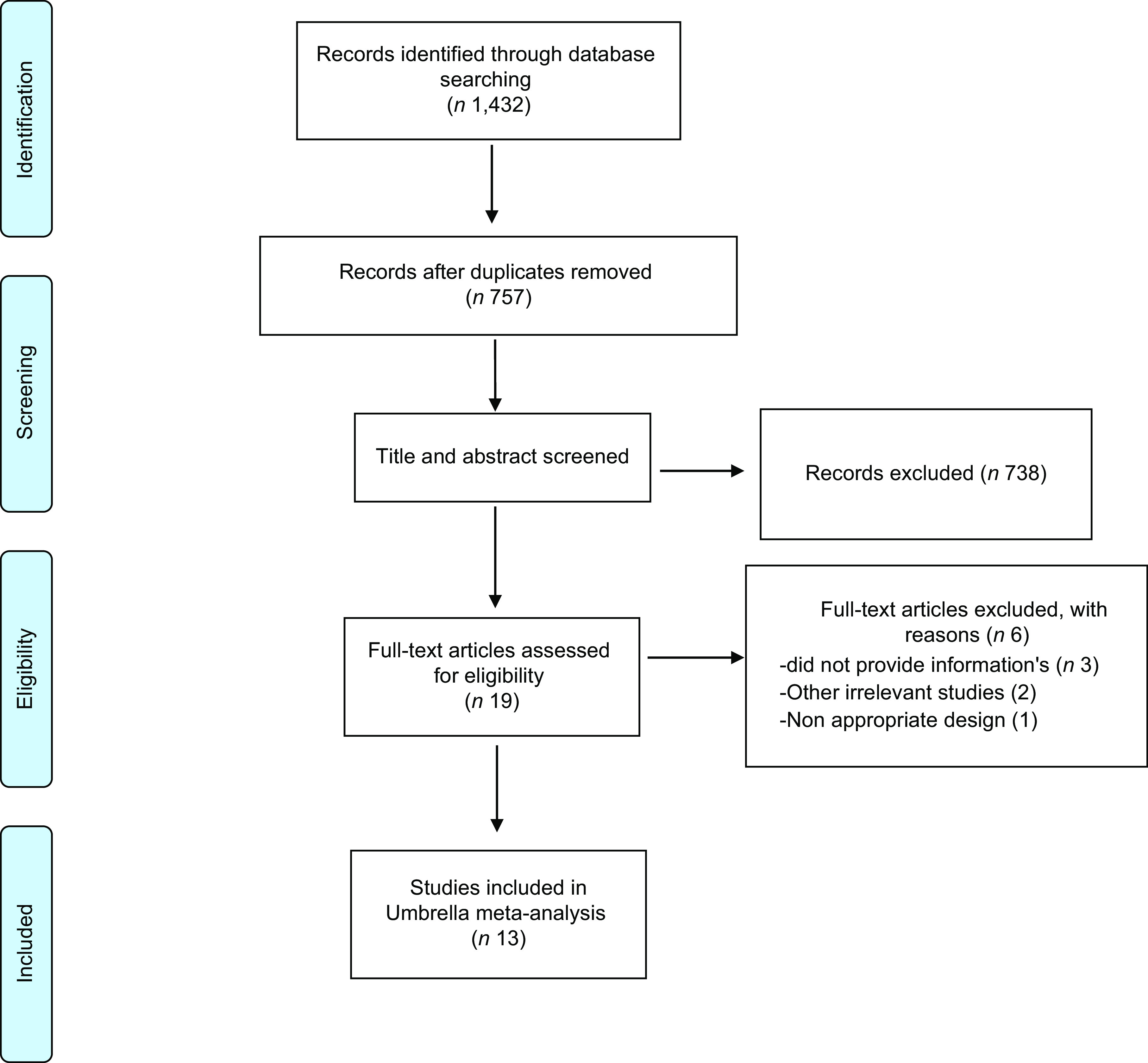
Preferred Reporting Items for Systematic Reviews and Meta-Analyses flow chart of the study

In initial search, a total of 1432 citations were identified. After discarding duplicates and screening of the remaining studies, of the 19 full texts, 13 meta-analyses summarising data from 4 randomised controlled trial (RCT) and 9 observational studies were included in the present analysis. The Preferred Reporting Items for Systematic Reviews and Meta-Analyses flow chart of the screening process is presented in Fig. 1. All included studies were published from 2019 to 2021. About 712 354 participants in observational studies and 4191 participants in experimental studies were included in this review. Observational studies were conducted in Iran^(17)^, Turkey^(4)^, China^(11)^, Brazil^(9)^, Ethiopia^(18)^, Ireland^(19)^, Lebanon^(15)^, Poland^(2)^ and USA^(20)^. Three of four experimental studies were conducted in India^(5,12,14)^ and one in Iran^(21)^. Calcifediol, cholecalciferol and calcitriol were types of vitamin D supplementation which used in experimental studies. Table 1 provides the details of characteristics of included observational and experimental studies reviewed.

**Table 1:** The characteristics of included meta-analyses

Citation (First author *et al.*, year)	Country	NO. of participants	No. of studies in meta-analysis	Age (year)	Primary outcome/intervention	Study types	Quality Assessment Scale
**Observational study**							
Kaya *et al.* 2021	Turkey	205 869	23	18–85	Occurrence of the risk of COVID-19 infection, severity and mortality	Case–control, cohort, cross-sectional	Yes (NOS)
Kazemi *et al.* 2021	Iran	9110	31	7–81	Association of vitamin D status with COVID-19 severity	Case–control, cohort, cross-sectional	Yes (NOS)
Pereira *et al.* 2020	Brazil	8176,	26	35–81	Vitamin D deficiency and COVID-19 severity	Retrospective, cohort, cross-sectional	Yes (RTI–Item Bank)
Teshome *et al.* 2021	Ethiopia	91 120	14	NR	COVID-19 infection	Case–control, cohort, cross-sectional	Yes (JBI tools)
Oscanoa *et al.* 2021	Ireland	2692	23	30–60	Association between 25-hydroxyvitamin D concentration and severe acute respiratory syndrome coronavirus-2 infection severity or mortality	Case–control, cohort, cross-sectional	Yes (NOS)
Bassatne *et al.* 2021	Lebanon	18 601	31	42–88	Mortality rate from COVID-19 infection	Case–control, cohort, cross-sectional	Yes (NOS)
Liu *et al.* 2021	China	361 934	10	18–81	Incident COVID-19	Case–control	Yes (NOS)
Szarpak *et al.* 2021	Poland	14 485	13	40–83	Incident COVID-19	Case–control, cohort	Yes (RoB 2 tool)
Munshi *et al.* 2020	USA	376	6	49–72	Association of vitamin D serum levels with COVID-19 severity and prognosis	Case–control, cohort	NR
**Interventional study**							
Nikniaz *et al.* 2021	Iran	259	4	47–88	Impact of vitamin D supplementation on mortality rate**/**calcifediol, cholecalciferol, calcitriol	Clinical trials, quasi-experimental, interventional pilot studies	Yes (JBI Critical Appraisal Tools)
Rawat *et al.* 2019	India	467	5	53–87	Impact of vitamin D supplementation on mortality rate and ICU admission**/**calcifediol, cholecalciferol, calcitriol	Clinical trials, quasi-experimental, interventional pilot studies	Yes (Cochrane)
Shah *et al.* 2021	India	532	3	NR	Impact of vitamin D supplementation on mortality rate and ICU admission**/**cholecalciferol, calcitriol	Retrospective case–control study, clinical trials	Yes (Cochrane)
Pal *et al.* 2021	India	2933	13	47–74	Impact of vitamin D supplementation on ICU admission**/**calcifediol, cholecalciferol	Quasi-experimental study, retrospective, observational study, prospective, observational study	Yes (Cochrane)

### Risk of bias assessment

Based on AMSTAR questionnaire, all included meta-analysis studies evaluated as good quality. The quality score of six out of 13 studies was 10 and 11, and the remaining studies scored 8 and 9. The results are presented in Table 2.

**Table 2:** Results of the assessment of the methodological quality of meta-analysis using AMSTAR questionnaire

Study	A priori design	Selection and data extraction	Literature search	Publication type	List of studies	Characteristics of the included studies	Assessed scientific quality	Scientific quality formulating conclusions	Methods used to combine the findings	Assessed publication bias	Conflict of interest stated	Quality score
Kaya *et al.* 2021	+	+	+	+	–	+	+	+	+	+	+	10
Kazemi *et al.* 2021	+	+	+	+	+	+	+	+	+	+	+	11
Pereira *et al.* 2020	+	+	+	–	–	+	+	+	+	+	–	8
Teshome *et al.* 2021	+	+	+	+	–	+	+	+	+	+	+	10
Oscanoa *et al.* 2021	+	+	+	–	–	+	+	+	+	+	–	8
Bassatne *et al.* 2021	+	+	+	+	–	+	+	+	+	+	+	10
Liu *et al.* 2021	+	+	+	+	–	+	+	+	+	+	+	10
Szarpak *et al.* 2021	+	+	+	–	–	+	+	+	+	+	–	8
Munshi *et al.* 2020	+	+	+	–	–	+	+	+	+	+	–	8
Nikniaz *et al.* 2021	+	+	+	+	–	+	+	+	+	+	+	10
Rawat *et al.* 2019	+	+	+	–	–	+	+	+	+	+	+	9
Shah *et al.* 2021	+	+	+	–	–	+	+	+	+	+	–	8
Pal *et al.* 2021	+	+	+	–	+	+	+	+	+	+	–	9

The result of assessing the methodological quality using AMSTAR: each item for included studies (? : can’t answer; – : means no; + : means yes).

### Meta-analyses on vitamin D and COVID-19 mortality

#### Interventional studies

**Fig. 2:**
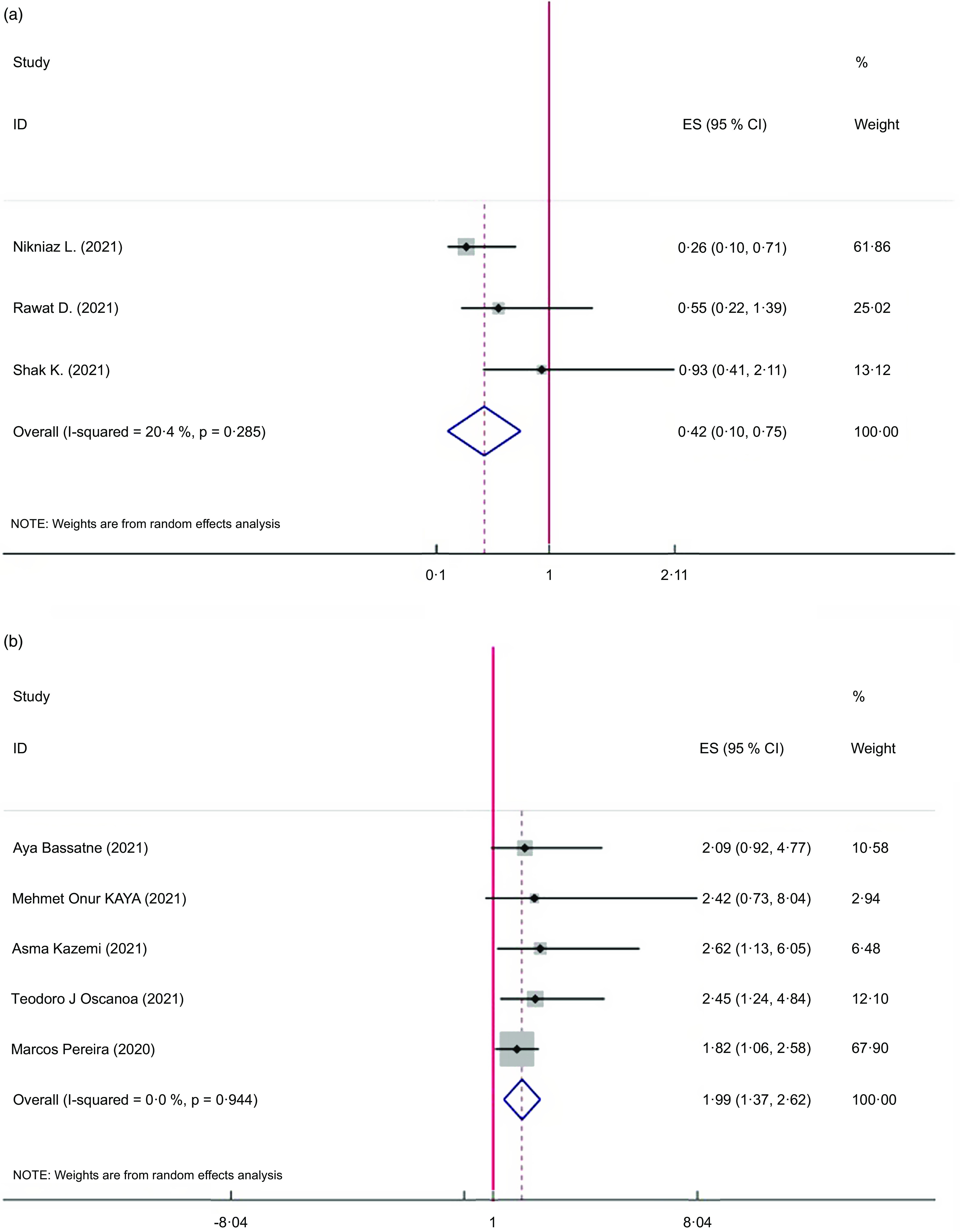
The Forest plot of umbrella meta-analysis on the effect of vitamin D supplementation on mortality according to interventional studies (a) and observational studies (b)

The pooled results of the 3 meta-analyses^(5,14,21)^ indicated that vitamin D supplementation significantly decreased mortality (ES = 0·42; 95 % CI: 0·10, 0·75, *P* < 0·001; *I* ^2^ = 20·4 %, *P* = 0·285). Sensitivity analysis showed that the removal of 1 study (Rawat *et al.*) affected the overall ES (ES = 0·47; 95 % CI: –0·13, 1·08) Fig. 2(a).

#### Observational studies

The results of the present umbrella meta-analysis from 5 studies indicated that vitamin D deficiency significantly increased mortality (ES = 1·99; 95 % CI: 1·37, 2·62, *P* < 0·001; *I* ^2^ = 00·0 %, *P* = 0·944) Fig. 2(b).

### Meta-analyses on serum vitamin D and COVID-19 positivity status

**Fig. 3:**
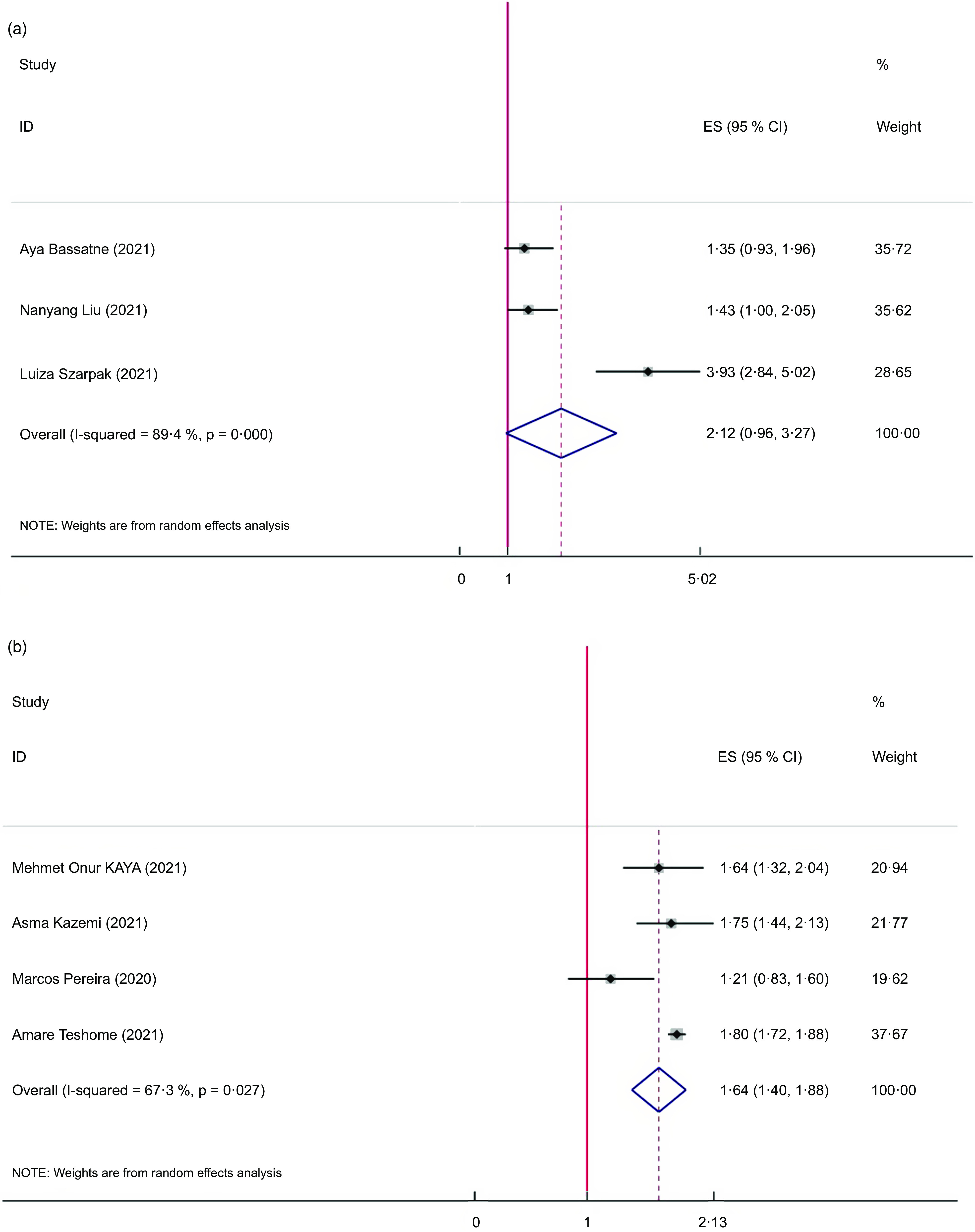
The Forest plot of umbrella meta-analysis on association of serum vitamin D with COVID-19 positivity status (a) and association of vitamin D deficiency with risk of infection in COVID-19 patients

The pooled results of the 3 meta-analyses did not show any significant relation between serum vitamin D and positive cases of COVID-19 (ES = 2·12; 95 % CI: 0·96, 3·27, *P* = 0·063; *I* ^2^ = 89·4 %, *P* < 0·001) (Fig. 3(a)).

### Meta-analyses on serum vitamin D deficiency and risk of infection in COVID-19 patients

Four meta-analyses were included in the analysis of the relation between vitamin D deficiency and risk of infection. Vitamin D deficiency significantly increased the risk of infection among COVID-19 patients (ES = 1·64; 95 % CI: 1·40, 1·88, *P* < 0·001; *I* ^2^ = 67·3 %, *P* = 0·027) (Fig. 3(b)).

### Meta-analyses on serum vitamin D and COVID-19 severity

**Fig. 4:**
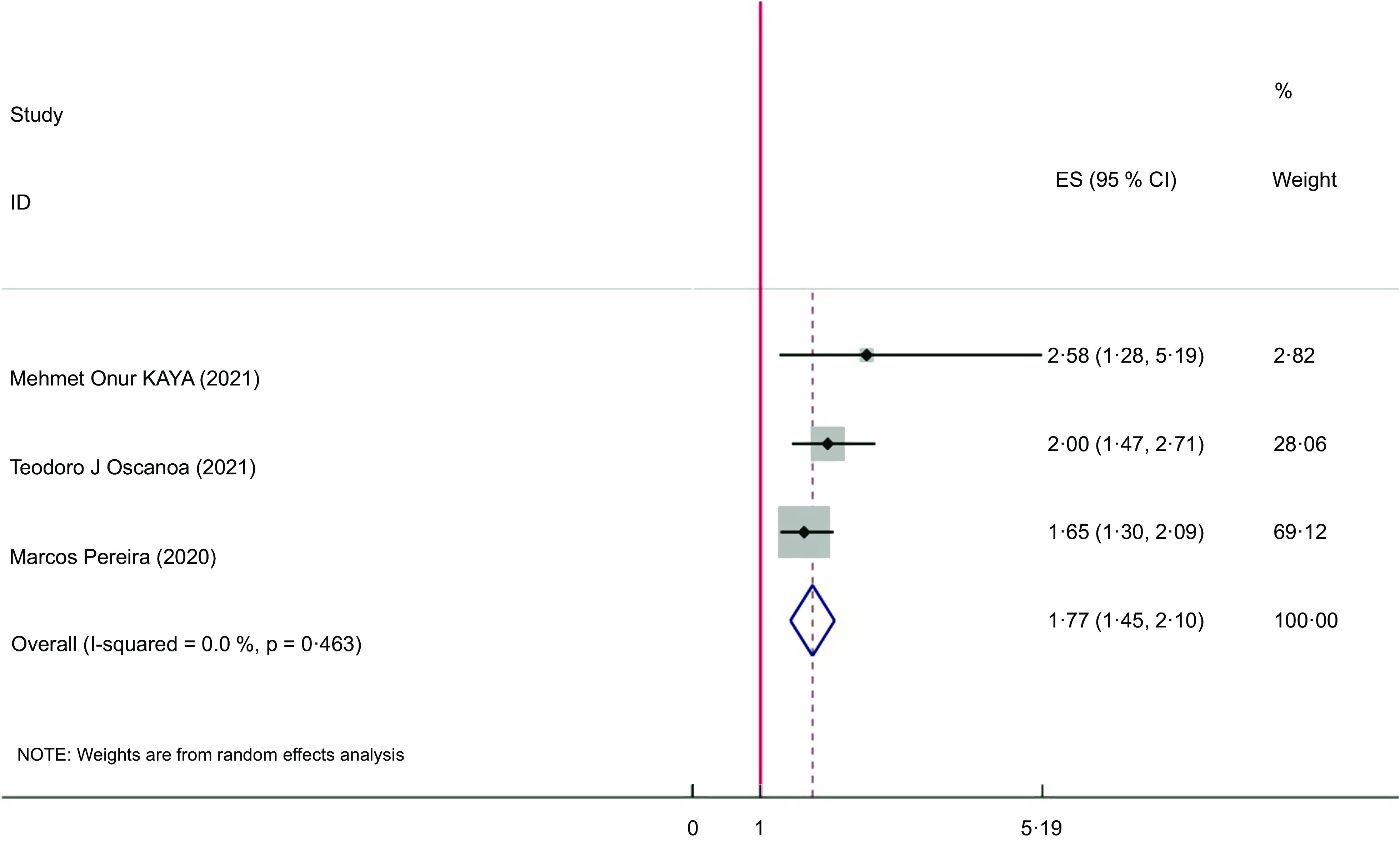
The Forest plot of umbrella meta-analysis on the association of vitamin D deficiency with COVID-19 severity

The pooled results of the 3 meta-analyses indicated a significant association between vitamin D deficiency and COVID-19 severity. Vitamin D deficiency increased the severity of COVID-19 (ES = 1·77; 95 % CI: 1·45, 2·10, *P* < 0·001; *I* ^2^ = 00·0 %, *P* = 0·463). Asma Kazemi *et al.’s* study was excluded from the analysis due to the wide CI and insignificant weight (weight = 0·02) (Fig. 4).

### Systematic reviews on vitamin D and other major health-related outcomes in COVID-19

Associations between vitamin D and ICU admission, mechanical ventilation and prognosis as the other health-related outcomes in COVID-19 have been reviewed in studies.

### Intensive care unit *admission*

Two review studies have assessed the impact of serum vitamin D status on ICU admission and severity of COVID-19. One study reported a positive but insignificant trend between vitamin D deficiency and increased risk of ICU admission^(15)^. The second study reported a high prevalence of vitamin D deficiency among severe COVID-19 cases compared to mild cases^(9)^. In another study, pooled analysis of unadjusted data from observational and RCT studies showed that vitamin D supplementation in COVID-19 was significantly associated with reduced ICU admission^(12)^. The results regarding ICU admission and vitamin D were contradictory in two systematic reviews of experimental studies: Rawat *et al.* found that vitamin D did not reduce ICU admission rates^(5)^, while Shah *et al.* reported lower ICU admission rate in patients supplemented with vitamin D compared to patients without supplementation^(14)^.

### Mechanical ventilation

Results regarding vitamin D and mechanical ventilation from two systematic review studies did not show any significant positive effect of vitamin D serum status or vitamin D supplementation on reducing risk of invasive, and non-invasive mechanical ventilation^(5,15)^.

### Poor prognosis

Finally, review of five studies revealed that patients with poor prognosis had significantly lower serum levels of vitamin D compared to those with good prognosis^(20)^.

## Discussion

The current umbrella meta-analysis summarises 13 meta-analyses, 57 observational studies and 23 RCT. According to results, vitamin D supplementation was efficient in reducing mortality, and vitamin D deficiency significantly increased mortality, severity of COVID-19, and risk of infection among patients. In addition, lower serum levels of vitamin D were significantly associated with poor prognosis. However, there was no significant relationship between serum vitamin D and positive cases of COVID-19, and the results regarding ICU admission and vitamin D were contradictory. Furthermore, results did not show any significant positive effect of vitamin D serum status or vitamin D supplementation on reducing risk of invasive or/and non-invasive mechanical ventilation. Due to limited number of studies for each variable, sub-group analyses were not possible.

In this umbrella meta-analysis, we discussed the multiple aspects of vitamin D deficiency and risk of mortality and COVID-19 health status outcomes. Vitamin D is a fat-soluble vitamin with anti-inflammatory, antioxidant and antiviral features^(21)^. The regulatory role of vitamin D on acquired and innate immunity explains its possible role in infectious diseases such as COVID-19^(18)^. Based on the findings of clinical trials, vitamin D supplementation is efficient in reducing mortality. The beneficial effects of vitamin D in treating COVID-19 are by preventing ‘cytokine storm’ and subsequent acute distress syndrome, known as the main cause of mortality^(22)^. After activation of the angiotensin-converting enzyme 2 (ACE2) receptor by the coronavirus, vitamin D provides its protective role via activating the renin–angiotensin–aldosterone system, modulating the cytokine storm and neutrophil activity, maintaining the pulmonary epithelial barrier, stimulating epithelial repair and reducing the damage caused by pro-inflammatory cytokines. Moreover, vitamin D augments the activity of the ACE2/Ang (1–7) axis, which has anti-inflammatory and antioxidant functions and also suppresses renin and the ACE/Ang II/AT1R axis, thereby enhancing the expression and concentration of ACE2, MasR and Ang-(1–7)^(5,15,23)^.

Vitamin D increases cathelicidin (LL-37)/defensin expression and displays antimicrobial and antiviral activities. Cathelicidin and defensin, furthermore, stimulate the expression of antiviral cytokines and chemokines involved in the recruitment of monocytes/macrophages, natural killer cells, neutrophils, and T cells and eventually enhance the host defence. The vitamin D receptor and CYP27B1 dignify the expression and cellular production of cathelicidin and defensin, which is effected by the interactions of pathogens and membrane pattern recognition receptors, including toll-like receptor and toll-like receptor 2^(4)^. Additionally, vitamin D indorses the up-regulation of IL-10 (anti-inflammatory cytokine) and down-regulation of IL-1, IL-6 (pro-inflammatory cytokines) and TNF-alpha^(12)^. Vitamin D also increases the expression of genes involved in the antioxidant system, such as the glutathione reductase gene^(17)^.

Although the majority of studies confirmed the efficiency of vitamin D supplementation in declining mortality, accurate evidence-based recommendations on circumstances of vitamin D administration in clinical practice can be confirmed by well-designed RCT on health outcomes of COVID-19^(15)^. In this regard, different aspects of vitamin D supplementation in COVID-19 in RCT must be discussed thoroughly. For example, some studies were accomplished on aged individuals which already have several comorbidities, are less exposed to sunlight, display lower 7-dehyrocholesterol values in the skin, have enhanced markers of cytokine release syndrome and are at high risk of respiratory failure^(2,9,21)^. Also, study population was not stratified based on serum vitamin D status at baseline, since vitamin D-deficient patients benefit more from supplementation. Differences in the dose of supplementation, frequency of supplementation, route of prescription and duration are other limiting factors^(21,23)^. Heterogeneity in the study design, population characteristics, methodology, baseline characteristics and small sample size of the population enrolled have also been mentioned in a number of studies^(5,14,21)^. Differences in the type and timing of vitamin D supplementation are another confounding factor. In regard to source of vitamin D, it has been mentioned that cholecalciferol supplementation may lead to faster recovery from COVID-19^(15)^. Most studies administered 1,25-hydroxy cholecalciferol, as the active form of vitamin D and few studies used calcifediol^(5)^. Moreover, one study indicated that patients supplemented with vitamin D after COVID-19 diagnosis benefited more than those supplemented with the drug prior to the diagnosis^(12)^.

**Fig. 5:**
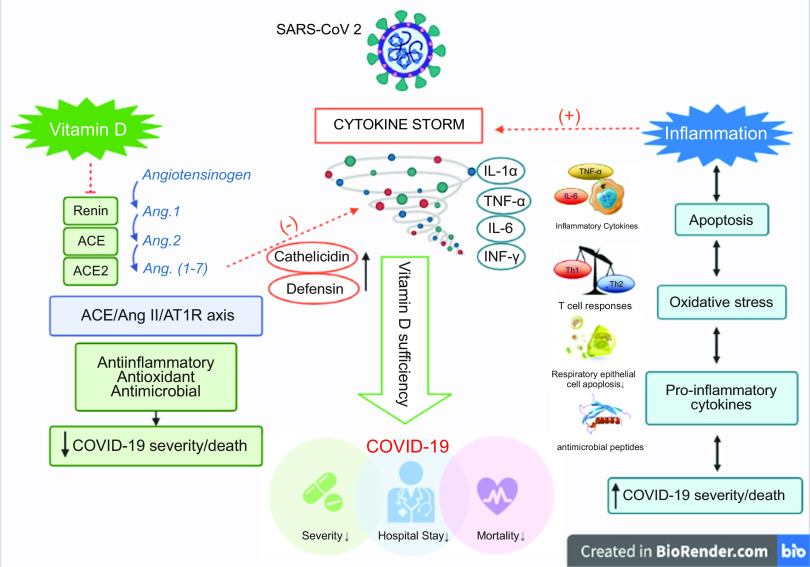
Mechanistic pathways demonstrating how vitamin D is affective on COVID-19 patients

According to observational studies, there was an inverse relationship between vitamin D deficiency and mortality. Vitamin D deficiency is related to reduced innate cellular immunity and cytokine storm stimulation^(11)^. The mechanism of action of vitamin D and ACE has been discussed earlier. High levels of ACE have been observed in patients with severe COVID-19 with low vitamin D level^(23)^. Vitamin D receptors are present on the nuclei membrane and are responsible for regulating different defensive proteins and receptors. Receptors recognise pathogens and their interaction affect the expression of pathogenic genes. Vitamin D inhibits T helper type 1 proliferation and shifts towards T helper type 2 proliferation, leading to decline in oxidative compounds synthesised via T helper type 1, affecting T-cell maturation, and producing anti-inflammatory subtypes^(21)^. McGregor *et al.* claimed that CD4 + T cells present in the bronchoalveolar lavage fluid of patients diagnosed with COVID-19 are Th1-skewed and the genes induced by severe acute respiratory syndrome coronavirus-2 are regulated by vitamin D receptor (VDR)^(24)^. Furthermore, vitamin D induces transcription factors including STAT3 (signal transducer and activator of transcription 3), c-JUN and BACH2 (BTB Domain And CNC Homolog 2) that cooperatively suppress Th1 and Th17, and eventually induce IL-10 via IL-6-STAT3 signalling^(23)^. Jain *et al.* reported that inflammatory markers such as IL-6, TNF-α and serum ferritin levels were shown low in COVID-19 patients with serum vitamin D level below 50 nmol/l^(25)^. Additionally, high concentrations of transforming growth factor *β* have been observed in the acute phases of COVID-19 and are relatively suppressed by VDR^(17)^. Mechanistic pathways are comprehensively and schematically demonstrated in Fig. 5.

The association between vitamin D deficiency and COVID-19 mortality must be discussed from other perspectives as well; for example, it is not clear whether low vitamin D is the cause or consequence of COVID-19. Multiple factors may affect the reduced vitamin D level in patients diagnosed with COVID-19, including age, sex, region, season, sun exposure, BMI, comorbidities and race. In favour of age, in the majority of studies, patients were over 50 years old with basic low vitamin D level^(11,15,21)^. Obesity alone is an independent risk factor for severe sequences of the disease^(2)^. COVID-19 broke out in winter when in the northern hemisphere, sunlight was low and individuals in that region had low 25-hydroxyvitamin D level^(4,11)^. Moreover, patients were enforced to be isolated or hospitalised, which prevented them from obtaining sunlight and a balanced diet^(11)^. Ecological studies have revealed that people living in higher latitude with decreased vitamin D level are prone to infection, related complications and mortality^(21)^.

In regard to studies, Liu *et al.* claimed inconsistency in the number and sample size of included studies, significant heterogeneity, publication bias and variations in ES estimates as reasons for the inconsistent results observed^(11)^. Bassatne *et al.* reported low quality and inevitability of evidence, as well as variation in the definition of vitamin D deficiency, serum 25(OH)D cut-offs and the timing of blood sampling and COVID-19 diagnosis and related outcomes in the included studies as the reason for the observed controversies among included studies. Also, decline in the synthesis of vitamin D binding protein and increase in 25(OH)D renal excretion which significantly regulate vitamin D level in critical illnesses could be considered as an additional explanation for discrepancies^(15)^.

Vitamin D deficiency also significantly enhanced the risk of COVID-19 infection and severity of COVID-19. According to the D-CIMA meta-analysis, patients with serum 25(OH)D < 20 ng/ml or 50 nmol/l were 1·64 times more likely to be infected with COVID-19 and also individuals with serum 25(OH)D < 20 ng/ml or 50 nmol/l were 2·42 times more likely to have severe COVID-19^(4)^. One study claimed that vitamin D supplementation declined the frequency of infection and was beneficial in patients receiving daily or weekly doses of 25(OH)D, protective effects were stronger in patients with baseline 25(OH)D less than 25nmol/l, and that this relationship was NS in those receiving bolus doses^(19)^. The mechanism of action is related to the disruption of the parathyroid-vitamin D-axis^(26)^. Moreover, vitamin D acts by stimulating the chemotaxis of T-lymphocytes and eliminating respiratory pathogens by inducing apoptosis and autophagy in the infected epithelium^(4)^. Hence, vitamin D declines the risk of microbial infection by modulating the innate and adaptive immunity, inhibiting cytokine storm, and declining pro-inflammatory cytokine production, due to its antiviral and anti-inflammatory properties^(17,18)^. Several aspects of this association must be further discussed. It is not clear whether the low concentrations of 25(OH)D in patients with severe COVID-19 infection are a cause or consequence of severe COVID-19 infection. Three perspectives have been mentioned: First, absence of baseline 25(OH)D measurement prior to infection; second, the concentration of C-reactive protein was not measured for patients with severe COVID-19 infection; third, 25(OH) D concentration decrease, as a consequence of inflammation, is considered solely as a negative acute phase reactant. Furthermore, a majority of studies did not report whether 25(OH)D concentrations were measured before or during COVID-19 infection^(19)^.

Patients with poor prognosis had significantly lower serum levels of vitamin D compared to those with good prognosis. One study claimed 25(OH)D concentration may be considered as a negative acute phase reactant and a poor prognosis in COVID-19 infection^(19)^. In Sun *et al.*’s study, 74 % of patients with severe COVID-19 had low calcium and 25(OH)D level and hypoproteinaemia. They reported hypocalcaemia as a biomarker of clinical severity and prognosis^(27)^. As mentioned earlier, calcitriol as the active form of vitamin D is the regulator of renin–angiotensin system and this overactivation is related to poor prognosis^(2)^.

According to the results of the present study, there was no significant relationship between serum vitamin D and positive cases of COVID-19. Bassatne *et al.* reported uncertain evidence regarding the association between positive cases of COVID-19 and serum 25(OH)D levels <20 ng/ml; however, increasing the cut-off of low 25(OH)D levels to 30 ng/ml showed significant results^(15)^. Other studies showed that COVID-19-positive cases had lower vitamin D level compared to negative cases. However, significant heterogeneity and publication bias was reported in these studies^(2,11)^.

The results regarding ICU admission and vitamin D were contradictory. Bassatne *et al.* claimed an increased risk of ICU admission in COVID-19 patients with 25(OH)D levels < 20 ng/ml and also indicated that calcifediol supplementation may have a protective effect on COVID-19-related ICU admissions^(15)^. Similarly, a pilot trial showed that only 1 out of 50 patients receiving calcifediol needed ICU admission, while 50 % of patients not receiving vitamin D were admitted to ICU (OR = 0·03). However, the reported OR was unreliable mainly due to indeterminate allocation concealment and patient blinding^(28)^. One study^(21)^ observed decline in ICU admission rate after vitamin D administration. However, this study did not include a RCT that had major influence on the findings of other studies which showed no association between ICU admission and vitamin D supplementation^(5)^. The main reason for the contradictory findings observed was the limited number of studies assessing the relationship between ICU admission and vitamin D.

The current study also did not show any significant positive effect of vitamin D serum status or vitamin D supplementation on reducing risk of invasive and non-invasive mechanical ventilation. One study showed that COVID-19 patients who required mechanical ventilation had at least one nutrient deficiency^(2)^. Hence, a clear association between vitamin D serum status and mechanical ventilation cannot be obtained. The main reason for the inconsistent results observed is the small number of studies assessing this association. The majority of studies did not observe any significant results, and the few ones lacked important methodological qualifications^(2,5,9,17)^.

## Strengths and limitations

The present study summarised the current evidences on the effects of vitamin D supplementation and deficiency in COVID-19 as the first umbrella meta-analysis. The current study was registered in PROSPERO or Cochrane Library and several aspects of COVID-19 health status outcomes were assessed. Based on the AMSTAR questionnaire, all included meta-analyses were evaluated as high quality. The limitations were the significant heterogeneity observed in few outcomes, and also, due to the limited number of studies, sub-group analysis was not possible. The novelty of the subject was in favour of the small number of studies included, especially RCT.

### Conclusion

The present umbrella of meta-analyses confirms the efficiency of vitamin D supplementation in reducing COVID-19 mortality. This review also indorses an inverse association between vitamin D deficiency and risk of mortality and infection among COVID-19 patients and the severity of COVID-19. In addition, lower serum levels of vitamin D were significantly associated with poor prognosis in patients. Hence, vitamin D supplementation is supported for preventing catastrophic outcomes of COVID-19.

## Supporting information

Jamilian et al. supplementary material Jamilian et al. supplementary material

## Supplementary material

For supplementary material accompanying this paper visit https://doi.org/10.1017/S1368980024000934

## References

[1] Liu F , Li L , Xu M et al. (2020) Prognostic value of interleukin-6, C-reactive protein, and procalcitonin in patients with COVID-19. J Clin Virol 127, 104370. 32344321 10.1016/j.jcv.2020.104370 PMC7194648

[2] Szarpak L , Rafique Z , Gasecka A et al. (2021) A systematic review and meta-analysis of effect of vitamin D levels on the incidence of COVID-19. Cardiol J 28, 647–654. 34308537 10.5603/CJ.a2021.0072 PMC8428943

[3] Daneshkhah A , Agrawal V , Eshein A et al. (2020) Evidence for possible association of vitamin D status with cytokine storm and unregulated inflammation in COVID-19 patients. Aging Clin Exp Res 32, 2141–2158. 32876941 10.1007/s40520-020-01677-y PMC7465887

[4] Kaya MO , Pamukçu E & Yakar B (2021) The role of vitamin D deficiency on COVID-19: a systematic review and meta-analysis of observational studies. Epidemiol Health 43, e2021074. 34607398 10.4178/epih.e2021074 PMC8769802

[5] Rawat D , Roy A , Maitra S et al. (2021) Vitamin D supplementation and COVID-19 treatment: a systematic review and meta-analysis. Diabetes Metab Syndrome: Clinical Research & Reviews 15, 102189. 10.1016/j.dsx.2021.102189 PMC8236412 34217144

[6] Martineau AR , Jolliffe DA , Hooper RL et al. (2017) Vitamin D supplementation to prevent acute respiratory tract infections: systematic review and meta-analysis of individual participant data. BMJ 356, i6583. 28202713 10.1136/bmj.i6583 PMC5310969

[7] Chakhtoura M , Napoli N & Fuleihan GEH (2020) Commentary: myths and facts on vitamin D amidst the COVID-19 pandemic. Metab Clin Exp 109, 154276. 32470350 10.1016/j.metabol.2020.154276 PMC7250097

[8] Grant WB , Lahore H , McDonnell SL et al. (2020) Evidence that vitamin D supplementation could reduce risk of influenza and COVID-19 infections and deaths. Nutrients 12, 988. 32492787 10.3390/nu12061620 PMC7352449

[9] Pereira M , Dantas Damascena A , Galvão Azevedo LM et al. (2022) Vitamin D deficiency aggravates COVID-19: systematic review and meta-analysis. Crit Rev Food Sci Nutr 62, 1308–1316. 33146028 10.1080/10408398.2020.1841090

[10] Pinzon RT & Pradana AW (2020) Vitamin D deficiency among patients with COVID-19: case series and recent literature review. Trop Med Health 48, 1–7. 33342439 10.1186/s41182-020-00277-w PMC7750008

[11] Liu N , Sun J , Wang X et al. (2021) Low vitamin D status is associated with coronavirus disease 2019 outcomes: a systematic review and meta-analysis. Int J Infect Dis 104, 58–64. 33401034 10.1016/j.ijid.2020.12.077 PMC7833186

[12] Pal R , Banerjee M , Bhadada S et al. (2021) Vitamin D supplementation and clinical outcomes in COVID-19: a systematic review and meta-analysis. J Endocrinological Invest 45, 53–68. 10.1007/s40618-021-01614-4 PMC8223190 34165766

[13] Murai IH , Fernandes AL , Sales LP et al. (2021) Effect of a single high dose of vitamin D3 on hospital length of stay in patients with moderate to severe COVID-19: a randomized clinical trial. Jama 325, 1053–1060. 33595634 10.1001/jama.2020.26848 PMC7890452

[14] Shah K , Saxena D & Mavalankar D (2021) Vitamin D supplementation, COVID-19 and disease severity: a meta-analysis. QJM: An International Journal of Medicine 114, 175–181. 33486522 10.1093/qjmed/hcab009 PMC7928587

[15] Bassatne A , Basbous M , Chakhtoura M et al. (2021) The link between COVID-19 and VItamin D (VIVID): a systematic review and meta-analysis. Metab 119, 154753. 10.1016/j.metabol.2021.154753 PMC7989070 33774074

[16] Shea BJ , Grimshaw JM , Wells GA et al. (2007) Development of AMSTAR: a measurement tool to assess the methodological quality of systematic reviews. BMC Med Res Method 7, 1–7. 10.1186/1471-2288-7-10 PMC1810543 17302989

[17] Kazemi A , Mohammadi V , Aghababaee SK et al. (2021) Association of vitamin D status with SARS-CoV-2 infection or COVID-19 severity: a systematic review and meta-analysis. Adv Nutr 12, 1636–1658. 33751020 10.1093/advances/nmab012 PMC7989595

[18] Teshome A , Adane A , Girma B et al. (2021) The impact of vitamin D level on COVID-19 infection: systematic review and meta-analysis. Front Public Health 9, 624559. 33748066 10.3389/fpubh.2021.624559 PMC7973108

[19] Oscanoa TJ , Amado J , Vidal X et al. (2021) The relationship between the severity and mortality of SARS-CoV-2 infection and 25-hydroxyvitamin D concentration—a metaanalysis. Adv Respir Med 89, 145–157. 33966262 10.5603/ARM.a2021.0037

[20] Munshi R , Hussein MH , Toraih EA et al. (2021) Vitamin D insufficiency as a potential culprit in critical COVID-19 patients. J Med Virol 93, 733–740. 32716073 10.1002/jmv.26360

[21] Nikniaz L , Akbarzadeh MA , Hosseinifard H et al. (2021) The impact of vitamin D supplementation on mortality rate and clinical outcomes of COVID-19 patients: a systematic review and meta-analysis. Pharm Sci 27, S1–S12.

[22] Maghbooli Z , Ebrahimi M , Shirvani A et al. (2020) Vitamin D sufficiency reduced risk for morbidity and mortality in COVID-19 patients. Available SSRN 3616008. Published online: 14 July 2020. doi: 10.2139/ssrn.3616008.

[23] Oristrell J , Oliva JC , Subirana I et al. (2021) Association of calcitriol supplementation with reduced COVID-19 mortality in patients with chronic kidney disease: a population-based study. Biomedicines 9, 509. 34063015 10.3390/biomedicines9050509 PMC8147982

[24] McGregor R , Chauss D , Freiwald T et al. (2020) An autocrine Vitamin D-driven Th1 shutdown program can be exploited for COVID-19. BioRxiv. Published online: 19 July 2020. doi: 10.1101/2020.07.18.210161.

[25] Jain A , Chaurasia R , Sengar NS et al. (2020) Analysis of vitamin D level among asymptomatic and critically ill COVID-19 patients and its correlation with inflammatory markers. Sci Rep 10, 1–8. 33214648 10.1038/s41598-020-77093-z PMC7677378

[26] Pizzini A , Aichner M , Sahanic S et al. (2020) Impact of vitamin D deficiency on COVID-19—a prospective analysis from the CovILD Registry. Nutrients 12, 2775. 32932831 10.3390/nu12092775 PMC7551662

[27] Sun J-K , Zhang W-H , Zou L et al. (2020) Serum calcium as a biomarker of clinical severity and prognosis in patients with coronavirus disease 2019. Aging (Albany NY) 12, 11287. 32589164 10.18632/aging.103526 PMC7343468

[28] Castillo ME , Costa LME , Barrios JMV et al. (2020) Effect of calcifediol treatment and best available therapy versus best available therapy on intensive care unit admission and mortality among patients hospitalized for COVID-19: a pilot randomized clinical study. J Steroid Biochem Mol Biol 203, 105751. 32871238 10.1016/j.jsbmb.2020.105751 PMC7456194

