# Supplementary material for: The role of vitamin D in outcomes of critical care in COVID-19 patients: evidence from an umbrella meta-analysis of interventional and observational studies
Source: Public Health Nutr. 2024 Apr 24;27(1):e127. doi: 10.1017/S1368980024000934 (PMC11112434; doi:10.1017/S1368980024000934)
Supplement: Jamilian et al. supplementary material [file S1368980024000934sup001.doc]

Supplementary table 1. Search strategy for databases

| **Groups** | **Descriptors** |
| --- | --- |
| Outcome | "COVID-19" |
| Exposure | “Vitamin D” |
| Setting | “meta” OR “meta-analysis”. |

**PUBMED**

| **Descriptors** |
| --- |
| ("vitamin d"[MeSH Terms] OR "Ergocalciferols"[MeSH Terms] OR "Cholecalciferol"[MeSH Terms] OR ("vitamin d"[Title/Abstract] OR "ergocalciferol"[Title/Abstract] OR "Cholecalciferol"[Title/Abstract])) AND ("COVID-19"[MeSH Terms] OR "SARS-CoV-2"[MeSH Terms] OR ("COVID-19"[Title/Abstract] OR "SARS-CoV-2"[Title/Abstract] OR "coronavirus disease"[Title/Abstract])) AND ("meta-analysis"[Publication Type] OR "meta-analysis"[Title/Abstract] OR "meta"[Title/Abstract]) |

**WEB OF SCIENCE**

| TS=(“vitamin D”) OR TS=(“ergocalciferol”) OR TS=(“cholecalciferol”) AND TS=(“COVID-19”) OR TS=(“coronavirsu disease”) OR TS=(“ SARS-CoV-2”) AND TS=(“ meta”) OR TS=(“ meta-analysis”) |
| --- |

**SCOPUS**

| **Descriptors** |
| --- |
| ( ( TITLE-ABS-KEY ( "Vitamin D" ) OR TITLE-ABS-KEY ( ergocalciferol ) OR TITLE-ABS-KEY ( cholecalciferol ) ) ) AND ( ( TITLE-ABS-KEY ( covid-19 ) OR TITLE-ABS-KEY ( sars-cov-2 ) OR TITLE-ABS-KEY ( "coronavirus disease" ) ) ) AND ( ( TITLE-ABS-KEY ( meta-analysis ) OR TITLE-ABS-KEY ( meta ) ) ) |

**EMBASE**

| **Descriptors** |
| --- |
| ("Vitamin D" OR "ergocalciferol" OR "cholecalciferol" AND (“coronavirus disease”) OR "COVID-19" OR "sars-cov-2") AND (“meta” OR “meta-analysis”) |

| Study | A priori design | selection and data extraction | literature search | publication type | list of studies | characteristics of the included studies | assessed scientific quality | scientific quality formulating conclusions | methods used to combine the findings | assessed  publication  bias | conflict  of interest  stated | Quality score |
| --- | --- | --- | --- | --- | --- | --- | --- | --- | --- | --- | --- | --- |
| Baker et al.2008 | + | - | + | ? | - | - | - | ? | + | + | - | 4 |
| Davis et al.2011 | + | - | ? | ? | - | - | - | ? | + | + | - | 3 |
| leach et al.2012 | ? | + | + | + | + | - | + | + | - | + | - | 7 |
| Akilen et al.2012 | + | - | + | + | + | + | + | ? | + | - | + | 8 |
| Allen et al.2013 | ? | - | - | + | + | - | - | ? | + | + | - | 5 |
| Ainehchi et al.2019 | + | + | + | + | - | - | ? | + | + | - | + | 7 |
| Deyno et al.2019 | + | + | + | + | + | - | + | + | + | + | + | 10 |
| Namazi et al.2019 | + | + | + | + | - | - | + | ? | + | + | + | 8 |
| Heydarpour et al.2020 | + | + | + | + | + | + | + | ? | + | + | + | 10 |
| Heshmati et al.2021 | + | + | + | + | + | - | - | ? | + | + | - | 7 |
| Kutbi et al 2021 | + | + | + | + | + | + | + | ? | + | + | + | 10 |

**Supplemental Table 1**: Results of assess the methodological quality of meta-analysis

The result of assess the methodological quality using AMSTAR: each item for included studies (? ; can't answer; *: Not applicable; - ; means no; +: means yes).
